# Inelastic response of silicon to shock compression

**DOI:** 10.1038/srep24211

**Published:** 2016-04-13

**Authors:** A. Higginbotham, P. G. Stubley, A. J. Comley, J. H. Eggert, J. M. Foster, D. H. Kalantar, D. McGonegle, S. Patel, L. J. Peacock, S. D. Rothman, R. F. Smith, M. J. Suggit, J. S. Wark

**Affiliations:** 1 Department of Physics, Clarendon Laboratory, University of Oxford, Parks Road, Oxford OX1 3PU, UK; 2 Atomic Weapons Establishment, Aldermaston, Reading, RG7 4PR, UK; 3 Lawrence Livermore National Laboratory, Livermore, CA 94551, USA

## Abstract

The elastic and inelastic response of [001] oriented silicon to laser compression has been a topic of considerable discussion for well over a decade, yet there has been little progress in understanding the basic behaviour of this apparently simple material. We present experimental x-ray diffraction data showing complex elastic strain profiles in laser compressed samples on nanosecond timescales. We also present molecular dynamics and elasticity code modelling which suggests that a pressure induced phase transition is the cause of the previously reported ‘anomalous’ elastic waves. Moreover, this interpretation allows for measurement of the kinetic timescales for transition. This model is also discussed in the wider context of reported deformation of silicon to rapid compression in the literature.

Single crystal silicon would appear to be an ideal test-bed for our understanding of materials response to dynamic compression. It is one of a small class of materials for which near perfect, well oriented, defect free, crystals can be obtained. As such, it has been the topic of study both experimentally, and computationally, for many decades. However, despite prolonged and repeated efforts to fully understand the mechanical response of silicon, there still exists considerable debate and disagreement in the literature as to how this apparently simple material deforms under high strain rate conditions.

Under low strain rate, near hydrostatic compression, Si has been shown to undergo a plethora of phase transitions from its familiar cubic diamond (cd) structure^1^,^2^. The material has been seen to transform to *β*-Sn at 12 GPa, and then to the closely related Imma and simple hexagonal (sh) structures at 13.2 and 15.4 GPa respectively. These transitions represent major structural and electronic modifications to the system. For example, the transition from cd to *β*-Sn is accompanied by a 24% volume collapse, and an onset of metallisation.

Gas gun based studies repeatedly show that for shock compression along [001], above a certain threshold pressure a multi-wave structure evolves. The nature of the second wave in shock compressed silicon has been a topic of considerable debate. Early interpretation of wave profile measurements assumed variously that the departure from an initially uniaxial (elastic) response was mediated by conventional plasticity^3^, or transition to one of silicon’s many high pressure phases^4^. More recent work utilising flash x-ray diffraction^5^,^6^,^7^ suggests that the three wave structure corresponds to initial elastic compression up to the Hugoniot Elastic Limit (HEL) of 9 GPa, followed by an unidentified inelastic response with large (greater than 3.5° full width) mosaic spread, of crystallites and finally a state with large volume collapse consistent with a complete phase transition to the high pressure sh phase. It should be noted that these results do not appear to rule out the possibility of mixed phase coexistence of *β*-Sn or Imma with the ambient cd phase in the ‘inelastic’ region. Other shock wave studies on similar length scales have focussed on electrical resistivity, reporting a sharp increase in conductivity at the HEL^8^. This result could also be consistent with a partial phase transition from semiconducting cd to the metallic *β*-Sn structure.

Laser driven shock experiments on nanosecond timescales have also been the focus of numerous studies. Smith *et al.* present results of velocimetry measurements exploring the rate dependence of the HEL which show that the leading elastic response and second wave could be understood in terms of Gilman model of plasticity^9^. Additionally, early work on *in-situ* diffraction from laser compressed solids took advantage of silicon’s highly crystalline nature to observe diffraction in both compression and release^10^,^11^. Of particular interest is the study of Loveridge-Smith *et al.*, who demonstrate an ‘anomalous’ two wave elastic response under [001] compression^12^, which was not reproduced by later gas gun work^7^. In addition, no diffraction signal from inelastically deformed material was identified. This is in stark contrast to numerous other experimental campaigns where both plasticity^13^,^14^,^15^,^16^ and phase transitions^14^,^17^,^18^,^19^ have been identified in other materials.

In this paper we will present the results of nanosecond white light Laue x-ray diffraction experiments which confirm the existence of ‘anomalous’ elastic response on length scales of relevance to laser compression experiments. In addition, these results show the onset of tension in the material. We will discuss this data in terms of phase transition based mechanism, with the support of molecular dynamics and elasticity code simulations, and demonstrate that once the pertinent length scales of experiments are taken into account, one can resolve the apparent discrepancies across the literature. In addition, the data provides important insight into the kinetics of the phase transition.

## Results

**Figure 1:**
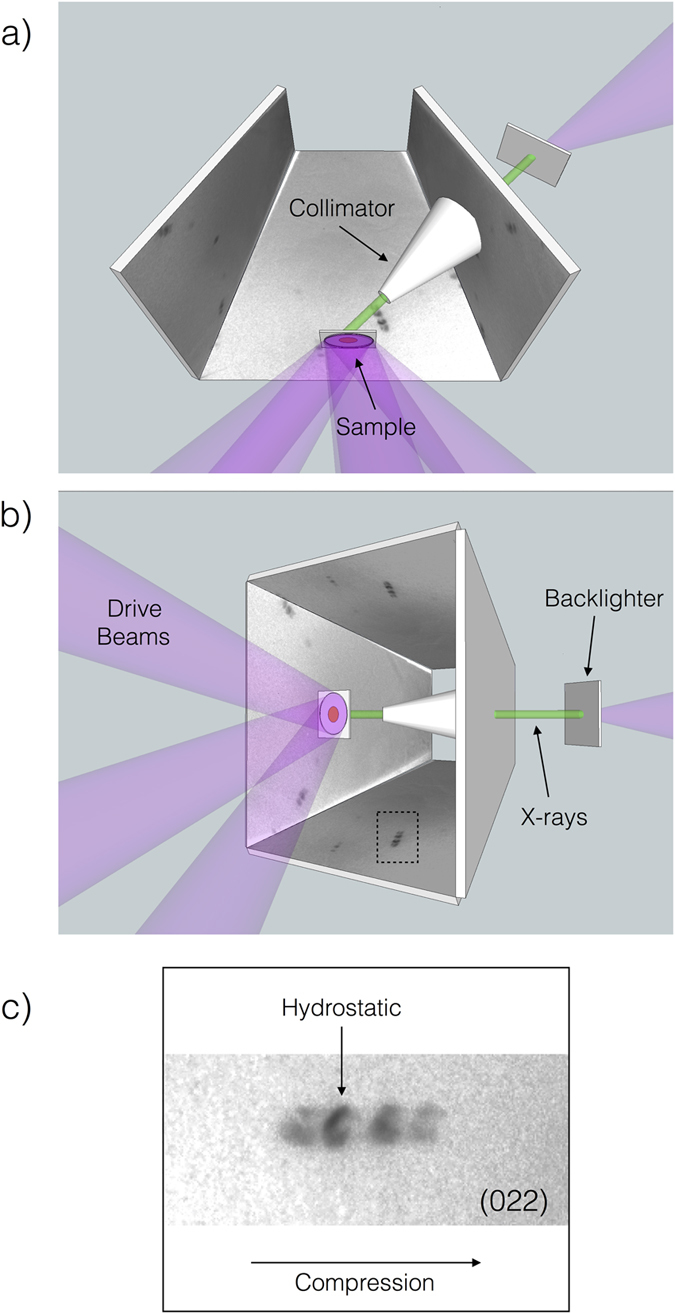
(**a**,**b**) Schematic of the experimental geometry from two angles. In both cases the base plate (which supports the sample) is omitted for clarity. The dashed box in (**b**) highlights a representative Laue peak, expanded in (**c**), showing the splitting of the feature consistent with the existence of one tensile, and two compressive strains.

Experiments to investigate the response of single crystal [001] silicon to shock compression were carried out on the ORION laser system at AWE, Aldermaston^20^. A schematic of the experimental setup is shown in Fig. 1. The sample was driven by five beams, each delivering 55–150 J of 351 nm light in a 5 ns square pulse. These beams were defocussed to 5 mm leading to a drive intensities of between 2.5–7 × 10^11^ Wcm^−2^. This overlap of beams, and significant defocus, allowed for a smooth drive across a 3 mm portion of the crystal.

The targets consisted of 30 *μ*m thick silicon with a 15 *μ*m parylene-N ablator and Al flash overcoat. The samples were seated in a truncated square pyramidal diagnostic, designed to allow recording of x-ray diffraction signal for almost the full 2*π* sr of reflection geometry solid angle. The inside of this diagnostic is lined with image plate to allow for recording of scattered x-rays. Note that although based on the previously reported BBXRD diagnostic^15^, this instrument has two key differences.

Firstly, the sample is mounted on the base of the pyramid, meaning that x-rays are incident on the image plate at closer to normal incidence. This affords more accurate registration of the position of the Laue spots on the image plate. Secondly x-rays are incident on the reverse (non-driven) side of the sample. This allows for the use of lower energy x-rays for white light studies. In this campaign, x-rays were generated using cocktail backlighter foils^21^,^22^. Foils are composed of 10 *μ*m PET support, overcoated with 600 nm layers of Gd, Sm, CsI, SnTe and Ag. Note that the Ti and V spectral tracers employed in early campaigns using these method are omitted here. Upon irradiation of the Ag side of the foil with a 1 ns square pulse of 351 nm light at 10^14 ^Wcm^−2^, the foils generate a quasi-continuous, albeit spectrally structured, x-ray spectrum from 3–10 keV. These x-rays were collimated to provide a 0.8 mm spot on the sample surface, aligned with the centre of the driven region. X-rays were timed so as to probe both driven and undriven material, typically 4–6 ns after the onset of drive.

As shown in Fig. 1, on compression, each Laue spot was observed to split into four. In addition to the original peak corresponding to uncompressed, hydrostatic material, two features are seen on the compression side, and a further one on the tension side. Since white light Laue diffraction is, in the absence of energy discrimination, sensitive only to cell aspect ratio, one cannot determine absolute strain from peak positions, only the aspect ratio change of the cell. However, the fact that peaks show no sign of broadening suggests minimal modification of crystalline microstructure, and is strongly indicative of a fully elastic response. In the case of the compressive features, this would imply strains of 6.5% and 12%, which lie within the range observed by Loveridge-Smith. It should be noted that these values were relatively consistent, varying by around ±1% between shots, and appear over the full range of drive strengths investigated in the experiment. This shot to shot variation is observed between shots with near identical drive conditions, and is not strongly correlated with drive strength, suggesting that the underlying process for the formation of these peaks may be stochastic in nature. It should be noted that these shot-to-shot variations in strain are far smaller than those observed by Loveridge-Smith. We attribute this to the more stable drive profile achievable in this experiment.

One further, and highly important deviation from the results of Loveridge-Smith is the appearance of the fourth feature, which under the assumption of elastic compression, would correspond to a 5% tension. Such tensile features have been observed in previous decaying wave experiments in silicon utilising short (1 ns) drive pulses^11^. One can understand the formation of regions of tension in these experiments as due to the interaction of two, counter propagating release waves, one due to reflection of the initial elastic shock front from the (free) rear surface, and the second due to release from the driven front surface due to removal of the supporting pressure as the drive laser switches off. However, for the 5 ns square pulses utilised in this study there is insufficient time for ablation surface release waves to interact with release from the target’s rear surface before x-ray probing. This is a key feature of the data as it suggests that a large amplitude release wave must be forming not at the drive surface, as is typically seen, but within the shock wave structure, and as such, must be associated with significant structural modification.

In order to gain a qualitative understand the origins of the complex elastic response, molecular dynamics simulations of shock compression along [001] Si were carried out. As with previously published work^23^, simulations were conducted using the LAMMPS code^24^, and the Erhart-Albe bond order potential^25^. A simulation of size 30 × 30 × 800 conventional cells (5,760,000 atoms) was thermalised to 300 K before being subjected to a 32 GPa shock. Unlike in previous work, this shock was launched by applying a constant force condition to a set of atoms forming a piston at the lower z boundary of the sample.

**Figure 2:**
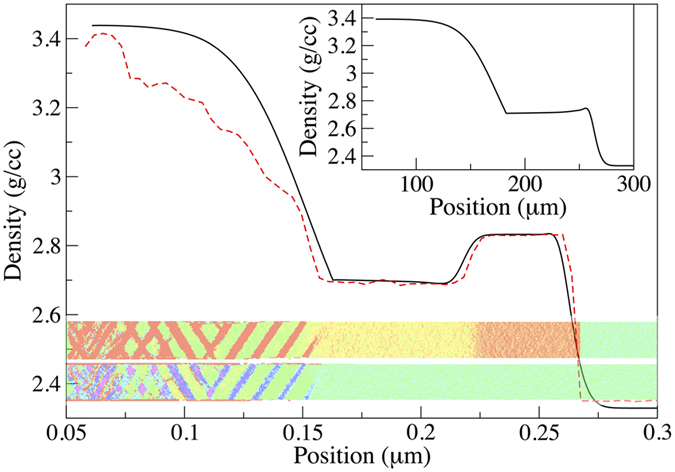
Density profiles for elasticity code (solid black) and MD (dashed red) for a 32 GPa shock in a 0.3 *μ*m sample. For reference, snapshots of the MD coded by the *F*_33_ (upper) and *F*_11_ (lower) per atom deformation gradient components are included. Shown inset (top right) is a elasticity based simulation showing the evolution of this profile out to a 300 *μ*m sample, where the distinct high strain plateau is absent.

The density profile at 30 ps after launching of the shock is shown in Fig. 2. Also shown are snapshots, colour coded by the transverse and longitudinal per-atom deformation gradient components^26^. Shown in the upper snapshot is the *F*_33_ component, corresponding to uniaxial compression along the shock direction. As previously reported, the banding structure towards the piston end of the sample is indicative of the phase transition from cd to Imma^23^. However, preceding this, two distinct regions of elastic compression are seen. The leading elastic wave has a strain of 20%, corresponding to an elastic response up to the initially applied 32 GPa longitudinal stress. This is followed by a release to a lower strain of 14.5%. The fully elastic nature of these regions is confirmed by the *F*_11_ component (the lower snapshot in Fig. 2), which remains at unity until the onset of the phase transition.

This two wave elastic behaviour is produced due to the large (~15%) volume collapse associated with the phase transition to Imma. As suggested above, the sample initially responds elastically, generating the leading high strain elastic (HSE) plateau. After 8 ps, the phase transition nucleates towards the piston. The small lag, indicative of some degree of kinetic inhibition, is key to the formation of the multi-wave elastic response, as release waves formed by volume collapse at the phase transition front are able to travel ahead of the transition, relaxing the material down into the low strain elastic (LSE) region. It is this release from HSE to LSE which will later lead to, and indeed is necessary for, the formation of tension when it interacts with the release from the free surface.

Note that similar effects of phase transition leading to partial release have been observed elsewhere in simulations of germanium undergoing shock compression, suggesting that although one may not be fully confident in the quantitative accuracy of MD simulations in this regime, a qualitative trend supportive of this two wave elastic response to phase transition is evident^27^.

One key observation relates to the morphology of the mixed phase region. As reported in earlier work^23^, the mixed phase consists of bands just tens of nanometres thick, and also exhibits large (several degree) bulk rotations of the cd and Imma phases in order to increase coherence at the boundaries between phases. The net effect is the production of highly broadened reciprocal space features which would most likely lead to diffraction signals too weak to be detected in white light or divergent beam geometries. As such, a mixed phase product of this type may explain the absence of diffraction from the inelastic region in reported laser based experiments.

Since MD simulations are limited in spatial and temporal scope, these two wave elastic phenomena were also incorporated into a Lagrangian Elasticity based code specifically designed to deal with materials with strength^28^. Although previous work based on hydrocodes has implemented a multi phase equation of state for silicon^29^,^30^, hydrocode models are typically not well suited to the study of predominantly elastic phenomena. The Lagrangian Elasticity code approach models the material as an elastic medium (as opposed to fluid), with dissipative mechanisms being added as distinct, additional models. This allows for the consistent modelling of mixed phase regions and kinetics, as well as a quantitative tracking of the material’s local deformation gradient.

In order to model the effects of phase transition, each cell in the simulation is allowed to transition to a mixed phase once the cell’s pressure has remained above a threshold (i.e. the phase transition pressure) for a defined period of time. This delay in the onset of phase transition mimics the kinetic inhibition discussed in the MD above. As a cell undergoes transition, its volume collapses. For cells in the middle of the simulation, this isolated, instantaneous collapse must be compensated for by a release of the neighbouring cells, ensuring that the overall sample length is conserved. It is this release towards the elastic region which results in the formation of a LSE region. The phase transition lag, rate of transformation, final phase fractions, as well as the pressure (and strength) dependent elastic constants in the low and high pressure phases are determined from MD. As in the MD, a constant 32 GPa stress is applied to one end of the simulation. The resulting density profile for a simulation on length and timescales commensurate with MD is shown in Fig. 2. Note that although the stochastic nature of the transition cannot be captured, the elasticity code recreates both the HSE and LSE regions.

There are a number of advantages to using the elasticity code approach to understand the two wave elastic response. Firstly, the MD suffers from an artificially high phase transition threshold of around 30 GPa (compared with a 13.2 GPa onset for hydrostatic compression in diamond anvil cell experiments^1^). Below this stress, simulations on reasonable time and length scales remain elastic. The onset at 30 GPa is associated with an instability in the potential, which lowers the barrier to transition to allow for Imma phase formation on picosecond timescales. By using the elasticity code, one can investigate the effect of lowering this phase transition pressure to levels commensurate with nanosecond experiments. Secondly, one can easily access much longer spatial and temporal scales, allowing for extrapolation of results between different experimental regimes. For example, in Fig. 2 we show the results of running the MD matched elasticity code out to 0.3 mm, where the LSE region has expanded to consume the HSE region. This suggests that any complex elastic behaviour resulting from a phase transition as seen in MD would be visible only in short timescale experiments, such as those driven by nanosecond (and shorter) laser-driven compression, and would be absent in gas gun experiments. Indeed, this is consistent with recent gas gun experiments conducted by Turneaure *et al.*^5^,^7^, where no signs of anomalous elastic response were identified.

**Figure 3:**
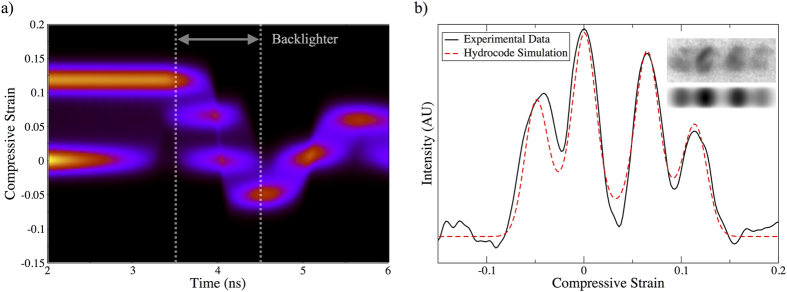
Elasticity code determined strain history the sample (**a**) after onset of the drive within the silicon. Shown inset in (**b**) are simulated and experimental white light Laue signals for x-ray exposure between 3.5–4.5 ns (as indicated by the dashed lines) in (**a**). This is consistent with the experimental backligher timing of 5 ns once transit time though the ablator is accounted for. Also shown in (**b**) are lineouts of these signals.

Since the elasticity code described above explicitly tracks longitudinal and transverse elastic strains, one can generate a simulated diffraction pattern for a given experimental geometry. In Fig. 3 we show the strain history of a simulation which has been optimised to match an experimentally observed response. This shot utilised a drive intensity of 4 × 10^11^ Wcm^−2^, with backlighter probing the sample 5 ns after the onset of drive at the ablator’s surface. This data is chosen as it displays the clearest distinction between peaks. Only strains within elastically compressed portions of the sample are considered, permitting comparison with the purely elastically response observed in diffraction. A summation of these strain profiles over a 1 ns window, assuming that the backlighter has spectrally flat response over the (300 eV) range of interest, and no significant energy dependence to sample reflectivity, allows us to create a simulated diffraction signal, as shown in 3b. This simulation clearly shows the formation of both HSE, LSE and tensile responses. Best fit to the experimental data is found for a lag time for phase transition of 1.2 ns. It should be noted that due the assumptions on spectral response discussed above, and the stochastic nature of the lag time between shots, this value should be taken as being representative. However, this significant kinetic lag is consistent with considerable predicted enthalpy barrier (of around 500 meV) between the cd and *β*-Sn phases^31^.

This reinforces the idea that this complex, phase transition driven, elastic behaviour is most likely a phenomena exclusive to laser based, nanosecond scale experiments. Moreover, this nanosecond scale kinetics is consistent with the artificially high threshold for phase transition in MD (which accesses only picosecond timescales).

## Discussion

It is pertinent to ask how these results compare to earlier laser work by Loveridge-Smith^12^. This paper describes a series of shots on the NOVA laser at LLNL. Samples were indirectly driven via VUV radiation from a laser irradiated hohlraum. Loveridge-Smith *et al.* report the LSE as preceding the HSE. Crucially, the drive history was not recorded, and it is reported that the strains measured were highly sensitive to the drive conditions. This is in contrast to the direct drive experiments reported here, where strains were consistent over a range of applied laser intensities. This disparity supports the conclusion in Loveridge-Smith’s work that the laser temporal profile may have been affecting sample response. Since one would expect that any variation of drive on the timescale of the kinetics (~1 ns) would significantly affect the model proposed above, this disparity in qualitative behaviour between these two experiments is not unexpected.

It should be noted that given the lack of diffraction from the inelastic region, we are unable to unambiguously determine whether a phase transition is indeed responsible. However, there are a number of reasons one could consider this mechanism a likely candidate. Firstly, as we have discussed, plasticity has been observed in diffraction for a wide range of materials, and yet no evidence of it is seen in this, or previous laser based experiments. Secondly, although the Gilman model of plasticity can reproduce a peak in strain at the HEL^9^, the parameters required to fit experimental wave speed profiles suggest initial defect densities larger than those measured for the sample, and defect velocities above the shear wave speed. This is in line with earlier arguments which suggest that plasticity may not become a competitive mechanism for shear relief in silicon on the short timescales accessible to laser experiments^12^. Most significantly, it is difficult to reconcile conventional plasticity mechanisms with such a sharply-defined two-strain elastic response, as seen in the data, especially in light of the need for a large volume collapse in order to explain the observed tensile feature.

One further observation is that the 6.5% strain observed in the LSE region, which can be interpreted as corresponding to the HEL, corresponds to a longitudinal stress of 10.7 GPa, below the hydrostatic transition pressure of 12 GPa^5^. This is consistent with density functional theory calculations which show a lowering of the cd → *β*-Sn transition pressure with increasing shear stress^31^. This may suggest that on longer timescales, phase transition, or a subsequent plastic response, may mediate response down towards the HEL of 9 GPa.

In summary, we have presented white light Laue data showing a complex elastic response of laser compressed [001] silicon. In order to explain observed tension we must invoke a model of material response which suggests that a phase transition to one of silicon’s many high pressure phases. By making use of an elasticity based Lagrangian code we are able to infer a kinetic timescale for these transitions, and in noting that this lies around 1 ns, we are able explain the apparently disparate conclusions of MD simulations, and laser and gas-gun driven experiments.

## Additional Information

**How to cite this article**: Higginbotham, A. *et al.* Inelastic response of silicon to shock compression. *Sci. Rep.* **6**, 24211; doi: 10.1038/srep24211 (2016).

## References

[b1] Mujica A., Rubio A., Muñoz A. & Needs R. J. High-pressure phases of group-IV, III–V, and II–VI compounds. Rev. Mod. Phys. 75, 863–912 (2003).

[b2] Ackland G. J. High-pressure phases of group IV and III–V semiconductors. Rep. Prog. Phys. 64, 483–516 (2001).

[b3] Pavlovskii M. N. Formation of metallic modifications of germanium and silicon under shock loading. Soviet Physics - Solid state 9, 2514–2518 (1968).

[b4] Gust W. H. & Royce E. B. Axial yield strengths and two successive phase transition stresses for crystalline silicon. J. Appl. Phys. 42, 1897 (1971).

[b5] Turneaure S. J. & Gupta Y. M. Inelastic deformation and phase transformation of shock compressed silicon single crystals. Appl. Phys. Lett. 91, 201913 (2007).

[b6] Turneaure S. J. & Gupta Y. M. X-ray diffraction and continuum measurements in silicon crystals shocked below the elastic limit. Appl. Phys. Lett. 90, 051905 (2007).

[b7] Turneaure S. J. & Gupta Y. M. Real-time x-ray diffraction at the impact surface of shocked crystals. J. Appl. Phys. 111, 026101 (2012).

[b8] Gilev S. D. & Trubachev A. M. Metallization of silicon in a shock wave: the metallization threshold and ultrahigh defect densities. J. Phys. Condens. Matter 16, 8139–8153 (2004).

[b9] Smith R. F. *et al.* Orientation and rate dependence in high strain-rate compression of single-crystal silicon. Phys. Rev. B: Condens. Matter Mater. Phys. 86, 245204 (2012).

[b10] Wark J. S., Whitlock R. R., Hauer A., Swain J. E. & Solone P. J. Shock launching in silicon studied with use of pulsed x-ray diffraction. Phys. Rev. B: Condens. Matter Mater. Phys. 35, 9391–9394 (1987). 10.1103/physrevb.35.9391 9941362

[b11] Wark J. S., Woolsey N. C. & Whitlock R. R. Novel measurements of high-dynamic crystal strength by picosecond x-ray diffraction. Appl. Phys. Lett. 61, 651 (1992).

[b12] Loveridge-Smith A. *et al.* Anomalous elastic response of silicon to uniaxial shock compression on nanosecond time scales. Phys. Rev. Lett. 86, 2349–2352 (2001). 11289926 10.1103/PhysRevLett.86.2349

[b13] Murphy W. J. *et al.* The strength of single crystal copper under uniaxial shock compression at 100 GPa. J. Phys. Condens. Matter 22, 065404 (2010). 21389369 10.1088/0953-8984/22/6/065404

[b14] Hawreliak J. *et al.* *In situ* x-ray diffraction measurements of the c/a ratio in the high-pressure *ε* phase of shock-compressed polycrystalline iron. Phys. Rev. B: Condens. Matter Mater. Phys. 83 (2011).

[b15] Comley A. J. *et al.* Strength of Shock-Loaded Single-Crystal tantalum [100] determined using *in situ* broadband X-Ray laue diffraction. Phys. Rev. Lett. 110, 115501 (2013). 25166552 10.1103/PhysRevLett.110.115501

[b16] Milathianaki D. *et al.* Femtosecond visualization of lattice dynamics in shock-compressed matter. Science 342, 220–223 (2013). 24115435 10.1126/science.1239566

[b17] Kalantar D. *et al.* *In situ* diffraction measurements of lattice response due to shock loading, including direct observation of the *α*–*ε* phase transition in iron. Int. J. Impact Eng. 33, 343–352 (2006).

[b18] Hawreliak J. *et al.* Analysis of the x-ray diffraction signal for the *α*–*ε* transition in shock-compressed iron: Simulation and experiment. Phys. Rev. B Condens. Matter 74, 16 (2006).

[b19] Coppari F. *et al.* Experimental evidence for a phase transition in magnesium oxide at exoplanet pressures. Nat. Geosci. 6, 926–929 (2013).

[b20] Hopps N. *et al.* Comprehensive description of the orion laser facility. Plasma Phys. Controlled Fusion 57, 064002 (2015).

[b21] Suggit M. *et al.* Nanosecond x-ray laue diffraction apparatus suitable for laser shock compression experiments. Rev. Sci. Instrum. 81, 083902 (2010). 20815613 10.1063/1.3455211

[b22] Suggit M. J. *et al.* Nanosecond white-light laue diffraction measurements of dislocation microstructure in shock-compressed single-crystal copper. Nat. Commun. 3, 1224 (2012). 23187624 10.1038/ncomms2225

[b23] Mogni G., Higginbotham A., Gaál-Nagy K., Park N. & Wark J. S. Molecular dynamics simulations of shock-compressed single-crystal silicon. Phys. Rev. B Condens. Matter 89, 064104 (2014).

[b24] Plimpton S. Fast parallel algorithms for short-range molecular dynamics. J. Comput. Phys. 117, 1–19 (1995).

[b25] Erhart P. & Albe K. Analytical potential for atomistic simulations of silicon, carbon, and silicon carbide. Phys. Rev. B: Condens. Matter Mater. Phys. 71, 035211 (2005).

[b26] Shimizu F., Ogata S. & Li J. Theory of shear banding in metallic glasses and molecular dynamics calculations. Mater. Trans. 48, 2923–2927 (2007).

[b27] Lane J. M. D. & Thompson A. P. Molecular dynamics simulation of shock-induced phase transition in germanium. In *Shock Compression of Condensed Matter* 2009: *Proceedings of the American Physical Society Topical Group on Shock Compression of Condensed Matter*, vol. 1195, 1157–1160 (AIP Publishing, 2009).

[b28] Wark J. S., Higginbotham A., Milathianaki D. & Gleason A. Combined hydrodynamic and diffraction simulations of femtosecond x-ray scattering from Laser-Shocked crystals. J. Phys. Conf. Ser. 500, 152016 (2014).

[b29] Swift D., Ackland G., Hauer A. & Kyrala G. First-principles equations of state for simulations of shock waves in silicon. Phys. Rev. B: Condens. Matter Mater. Phys. 64, 214107 (2001).

[b30] Heuze O. & Swift D. Analysis and modeling of laser ramps and shocks in tiitatium and zirconium with phase transitions. In *Shock Compression of Condensed Matter 2011: Proceedings of the Conference of the American Physical Society Topical Group on Shock Compression of Condensed Matter*, vol. 1426, 1541–1544 (AIP Publishing, 2012).

[b31] Gaál-Nagy K. & Strauch D. Transition pressures and enthalpy barriers for the cubic diamond–*β*-tin transition in si and ge under nonhydrostatic conditions. Phys. Rev. B: Condens. Matter Mater. Phys. 73, 134101 (2006).

